# Psychological and Behavioral Contributors to Lower Urinary Tract Dysfunction: Trauma, Stress, and Neurobiological Pathways

**DOI:** 10.1007/s11884-026-00824-x

**Published:** 2026-08-19

**Authors:** Alexandra Neumann, Julianna Longo, Elise J. B. De, Andrew Schrepf, Anna Grace Kelly, Lindsey C. McKernan

**Affiliations:** 1 Faculty of Medicine, Semmelweis University, Üllői út 26, Budapest, 1085 Hungary; 2 Department of Psychiatry & Behavioral Sciences, Vanderbilt University School of Medicine, 47 New Scotland Avenue, Albany, NY 12208 USA; 3 Department of Urology, Albany Medical College, 3401 West End Ave #380, Nashville, TN 37203 USA; 4 Department of Anesthesiology, University of Michigan Medical School, Ann Arbor, USA; 5 Department of Psychology, George Mason University, 4400 University Drive, Fairfax, VA 22030 USA; 6 Department of Physical Medicine & Rehabilitation, Vanderbilt University School of Medicine, 3401 West End Ave #380, Nashville, TN 37203 USA; 7 Department of Urology, Vanderbilt University Medical Center Affiliation Postal Addresses, 3401 West End Ave #380, Nashville, TN 37203 USA; 8 Osher Center for Integrative Health at Vanderbilt, 3401 West End Avenue, Suite 380, Nashville, TN 37203 USA

**Keywords:** Voiding dysfunction, Inflammation, Trauma, Neurobiology, Stress, Chronic pelvic pain, Interstitial cystitis

## Abstract

**Purpose of Review:**

Chronic pelvic pain and lower urinary tract dysfunction are associated with a high prevalence of depression, anxiety, trauma history, and stress-related disorders, with worsening of symptoms in these groups. This review characterizes the mechanisms behind these associations and the benefits of intervention.

**Recent Findings:**

Trauma, stress, and neurobiological pathways interact as psychological and behavioral contributors to lower urinary tract symptoms and pelvic pain. The immune system and the neuraxis share pathways in chronic pain as it relates to stress regulation in urologic populations, with implications for symptom presentation. Trauma, including early life adverse experiences and later diverse stressors, can potentiate these pathways.

**Summary:**

Co-occurrence of psychological factors with incontinence, retention, urinary urgency, frequency, nocturia, and bladder pain is common and clinically relevant to the urologist. Psychological interventions have been demonstrated to improve both quality of life and the symptoms themselves in concert with standard organ-based treatment approaches.

## Introduction

Lower urinary tract symptoms (LUTS) may originate from the bladder, prostate, urethra, and/or adjacent pelvic floor or pelvic organs, or may be referred from similarly innervated structures [1]. LUTS encompass storage and voiding symptoms, incomplete emptying, and lower urinary tract pain, including bladder or urethral pain, or pain associated with bladder filling/voiding [2]. Chronic pelvic pain (CPP), defined by the European Association of Urology as “chronic or persistent pain perceived* in structures related to the pelvis of either males or females...often associated with negative cognitive, behavioural, sexual and emotional consequences, and symptoms suggestive of lower urinary tract (LUT), sexual, bowel, pelvic floor or gynaecological dysfunction” [3]. Syndromes in urology associated with CPP and LUTS include interstitial cystitis/bladder pain syndrome (IC/BPS) and chronic prostatitis/chronic pelvic pain syndrome (CP/CPPS), which collectively fall under the umbrella term urologic chronic pelvic pain syndrome (UCPPS) according to the Multidisciplinary Approach to the Study of Chronic Pelvic Pain (MAPP) Research Network definition [4].

Individuals with LUTS and CPP experience high rates of psychosocial distress, which can worsen symptoms, quality of life, and prognosis [5–7]. Symptom presentations are often diverse and highly variable. For this reason, multidisciplinary and tailored care is often recommended as a treatment approach, as organ-based clinical focus, which seeks to identify a single anatomical source behind symptoms, fails to account for patient heterogeneity and psychosocial factors contributing to symptoms. Extensive efforts have been undertaken to better understand the different phenotypic presentations contributing to LUTS and CPP, including the role of psychosocial comorbidities in presentation.

Despite expanding knowledge of the relationship between psychosocial factors and LUTS, major gaps remain in clinical practice. Current diagnostic and treatment guidelines for LUTS focus primarily on biomedical markers, medications, and procedural interventions, often underemphasizing the role of trauma, stress, or psychological comorbidities contributing to symptoms [8, 9]. Emerging research, including work from the Symptoms of Lower Urinary Tract Dysfunction Research and Multidisciplinary Study of the Approach to Pelvic Pain Networks (LURN/MAPP), explicitly highlights non-urological factors as key contributors to condition subtypes, or “phenotypes,” with potential treatment implications [8–12]. However, in practice, urologists are left trying to integrate physical, neurological, psychosocial factors during short, bladder-focused consultations, often without sufficient resources to comprehensively address and distill this information into clinical dialogue.

The primary focus of the review is to detail recent information regarding psychosocial comorbidity in LUTS/CPP, neurobiological stress pathways and phenotyping, and to translate research findings to clinical practice. In addition to exploring current literature, we emphasize strategies for identification and effective patient-centered communication regarding psychosocial distress, and provide guidance on appropriate referral pathways and treatment offerings to support multidisciplinary care.

## Psychological and Behavioral Contributors to LUTS and CPP

Psychological factors, such as anxiety, depression, and, according to more recent research, obsessive-compulsive disorder (OCD), are prevalent and contribute to symptom burden in LUTS and CPP [12–15]. Evidence indicates a bidirectional relationship in which psychological distress can both precede and perpetuate urinary symptoms and pain [13, 16]. Distress can also develop in response to symptom onset [17]. Beyond symptom exacerbation, psychosocial comorbidity can impact bladder function. For example, people with comorbid OCD have higher rates of bladder outlet obstruction than individuals with LUTS alone (i.e. 45% vs. 17%, *p*=0.001), and 62% exhibit abnormal urodynamic studies [18]. Importantly, psychosocial distress often occurs in the broader context of trauma exposure and chronic hyperarousal, with trauma-related sequelae increasingly recognized as clinically relevant comorbidity.

Traumatic experiences occur at high rates among individuals with LUTS, CPP, and UCPPS and are consistently associated with increased symptom burden. Data from LURN demonstrates that 69% of patients with LUTS reported a history of childhood trauma, with a substantial proportion experiencing childhood sexual trauma [e.g. 25% of women in the sample] [19]. Notably, childhood sexual trauma was associated with greater incontinence severity in adulthood, highlighting the potential for early life experiences to shape long-term urinary function [19].

Posttraumatic stress disorder (PTSD) can develop in response to trauma exposure in a portion of patients. Hallmark symptoms of PTSD include chronic hyperarousal and re-experiencing of events [20]. Evidence suggests that PTSD is more common in LUTS/CPP (e.g. 22–42%), and when present, exacerbates symptoms beyond the impact of the trauma exposure itself [5, 21]. For example, trauma exposures, including intimate partner violence in adulthood, can heighten risk for stress and urge incontinence and nocturia, with particularly strong associations observed in individuals with exposure to partner violence and PTSD symptoms (e.g. nocturia OR increasing to 1.95), mirroring findings suggesting that PTSD is associated with more complex and severe symptom presentations [5, 21, 22].

Importantly, associations between trauma and LUTS persist after accounting for PTSD symptomology. In a companion study to the Reproductive Risks of Incontinence Study at Kaiser (RRISK) cohort, Raphael et al. (2022) found that history of sexual assault was independently associated with bladder pain (OR 1.39) [23]. Roberts et al. found that military sexual trauma was independently associated with increases in voiding issues, storage difficulties, IC/BPS, and fecal incontinence, suggesting a strong correlation between physical/emotional trauma exposure and impacted lower urinary tract and pelvic floors of patients [24].

Taken together, this recent research emphasizes the complexity of psychosocial distress experienced by many patients presenting with dysfunctional voiding and pelvic pain. Research is now turning toward understanding why and how trauma exposure and stress reactivity can impact pelvic physiology, and their relation to our understanding of phenotypic presentations of LUTS and CPP.

## Neurobiological Pathways and Stress

The National Institute of Health supported second study phases of both the LURN and MAPP Networks to examine underlying mechanisms influencing different patient subgroups in CPP and LUTS as a part of their core scientific mission(s) [4, 8, 25]. Both groups placed a major emphasis on understanding how non-urologic associated symptoms, particularly centralized pain and psychosocial distress, contribute to symptom profiles and response to standard therapies [4, 8, 25].

### Widespread Pain, Sensory Sensitivity, and Nociplastic Phenotyping

A foundational finding from the MAPP network’s initial study was that approximately two-thirds of participants had pain outside the pelvis, suggesting broader dysregulation of pain processing through concepts like central sensitization or allied terms (nociplastic pain) [4]. The MAPP Research Network Symptom Pattern Study (SPS) was a longitudinal observational study that followed MAPP-I and included deep phenotyping elements: brain imaging, sensory testing, immune markers, and extensive self-report symptom data from individuals with UCPPS [25, 26]. Study findings have helped the urologic community better understand how nociplastic pain impacts UCPPS.

In 2020, the International Association for the Study of Pain (IASP) defined three main mechanistic categories of chronic pain: nociceptive, neuropathic, and nociplastic pain [27]. Nociplastic pain refers to pain arising from altered nociception despite no clear evidence of actual or threatened tissue damage causing the activation of peripheral nociceptors or evidence for somatosensory disease involvement [27, 28].

According to a recent report (2023), MAPP-II found that 49% of participants had a nociplastic component to their presentation [9]. Importantly, pain phenotype appears stable over time for the vast majority of patients [5, 29]. The clinical hallmarks of nociplastic pain include widespread (pain affecting multiple regions on the body simultaneously) and migratory pain (pain that shifts from one location to another over time), coupled with cognitive difficulties (thinking and remembering), low energy, sleep disturbances, and environmental sensitivity (e.g., sensitivity to lights, sounds, or multiple sensory modalities), the latter of which increasingly tracks with the nociplastic phenotype across chronic pain conditions [28, 30].

Nociplastic pain is therefore strongly associated with the concept of Chronic Overlapping Pain Conditions (COPCs), a subset of common chronic pain conditions that are co-morbid with one-another such as fibromyalgia, irritable bowel syndrome, and interstitial cystitis [9, 31]. This symptom presentation can be remembered by the acronym SPACE: *S*leep disturbance, widespread and migratory *P*ain, *A*ffective distress, *C*ognitive depletion, and reduced *E*nergy levels [32]. Individuals with nociplastic pain also tend to show more sensitivity to experimental pain, and present with increased psychological complexity and symptom severity [28, 30]. PTSD is associated with nociplastic presentations in UCPPS. A recent study found that approximately 40% of IC/BPS patients met criteria for active PTSD at the time of initial consultation, and all patients with PTSD comorbidity had widespread pain.

LURN-II (2026) found evidence of central sensitization in 21% of individuals with urinary urgency, aligning with the premise that altered afferent signaling can be present in both pain and non-pain related presentations of LUTS [29, 33]. More recently, a composite measure of Generalized Sensory Sensitivity including interoceptive sensitivity, exteroceptive sensitivity, and widespread pain was examined in a large cohort of individuals with LUTS [29]. The authors found that people with LUTS who experienced urinary urgency and worse health-related quality of life had higher levels of multisensory sensitivity and widespread pain, suggesting that some LUTS cases implicate global sensory dysregulation [29].

### Clinical Utility of Simple Phenotyping Measures

The MAPP research network showed that common pain mechanisms are not the same for everyone with urinary pain [28]. Using simple self-report measures to classify patients into nociceptive, neuropathic, and nociplastic pain, MAPP demonstrated that individuals with neuropathic and nociplastic pain mechanism features showed the highest levels of pain interference and restriction of activities [9, 27]. These findings suggest that simple phenotyping tools can differentiate participants both for overall disease burden, and for specific points of emphasis, e.g., higher neuropathic pain was associated with greater sensitivity on pelvic exam [31].

The clinical relevance of these findings warrants emphasis, as emerging data strongly suggest that patient phenotype can be reliably identified using self-report measures and influence treatment trajectories. Using a brief self-report measure of widespread pain, environmental sensitivity, and internal sensitivity — Schrepf, As-Sanie, and colleagues (2021) demonstrated that pre-surgical scores predicted failure to achieve 50% pain reduction after hysterectomy for CPP, with an area under the curve exceeding 0.80 [31, 34]. This simple, short measure (the GAUGE) outperformed depression and anxiety scores in predicting persistent postoperative pain [35].

The presence of widespread pain may also impact treatment response in UCPPS. In following a longitudinal cohort through treatment, MAPP found that patients with widespread pain improved significantly with centrally-directed therapies (e.g. central nervous system stimulation focused treatments such as tricyclics or gabapentinoids), while those with localized pain showed no average improvement with the same medications (interaction *p* = 0.005) [36]. Conversely, patients with localized pain responded preferentially to peripherally-directed therapies such as pelvic floor physical therapy and cystoscopy with hydrodistention [36]. This finding is echoed by a critical (2025) reanalysis of RCTs for common treatments for IC/BPS by widespread pain stratification: participants with low levels of widespread pain responded more favorably to treatments directed at local symptoms (e.g., pentosan polysulfate and hydroxyzine) [37].

### Immune Sensitization: The TLR4 Pathway

In order to understand the immune response across phenotypes, MAPP conducted extensive assessments employing an immunoreactivity assay in which peripheral blood mononuclear cells (PBMCs) or whole blood underwent ex vivo stimulation [38, 39]. In both the Epidemiology Phenotyping Study (EPS) and SPS MAPP studies, higher immunoreactivity was associated with more widespread pain and the presence of comorbid pain syndromes [25, 38, 39]. Those with higher TLR immunoreactivity at the beginning of the EPS showed less naturalistic improvement over the following year [33]. In a study of the effects of *chronic* immunoreactivity, participants who showed exaggerated immune responses at all three timepoints across 18 months were compared to those whose response was consistently low [40]. Participants with high immunoreactivity were more sensitive to experimental pain at multiple body sites, confirming a systemic rather than organ-specific process [40].

These findings indicate that chronic high immunoreactivity can manifest as whole-body pain sensitivity. Clinically, the distinction was readily apparent on body maps, but the most striking finding was that participants with high immunoreactivity used *2.5 times as much healthcare* — measured by medication changes, physician calls, office visits, and procedures [40]. This finding parallels recent insurance data demonstrating that overlapping pelvic symptomatology is common and costly [41], and a particular issue for patients with CPP [41, 42]. Thus, there is potential clinical and economic value in early phenotypic assessments to prospectively match patients with better care options.

### Stress, Trauma, and Immune Priming

These neurobiological features of nociplastic pain are strongly associated with the magnitude of both recent and early life stress [43, 44]. When assessed, UCPPS populations almost invariably show high levels of trauma exposure, and patients frequently report that their symptoms are exacerbated by recent stress [5, 43]. The MAPP Network confirmed that the TLR4 immunoreactivity pathway is associated with trauma exposure in its participants [43]. The interaction between early and recent stress proved particularly important: individuals with IC/BPS who reported both life-threatening childhood trauma and major stress in the preceding six months endorsed approximately 16 pain sites on the body map, compared to roughly six in any other combination of stress categories [45].

This amplification of symptoms when early and recent adversity co-occur suggests that the stress sensitization pathways studied by psychologists and the neurobiological pathways identified through immune and brain imaging research converge on a common mechanism that may represent an actionable therapeutic target [46, 47].

### Implications for Phenotypic-Based Assessments

Phenotyping approaches are commonly used in urologic consultation to organize the complex interplay of heterogeneous symptoms and co-morbidities often present in CPP and LUTS. Current models emphasize both urologic and non-urologic symptoms that contribute to a person’s presentation (i.e. UPOINT/INPUT) [48, 49]. Recent findings from LURN and MAPP demonstrate that straightforward clinical assessment of pain distribution, stress sensitivity, and trauma exposure can be integrated into phenotypic-based assessment approaches to provide potentially actionable information for treatment selection and patient education [9, 29, 36]. Note that these approaches are complementary and not mutually exclusive [50]. Many patients have more than one phenotypic driver of symptoms, and phenotyping should occur alongside investigation of the extensive systemic, neurologic, and structural pathology that can contribute to symptoms [50, 51]. Thus, we recommend that these factors be integrated into a comprehensive screening and assessment process.

To assess nociplastic pain, we suggest supplementing existing phenotyping approaches with measures such as the Generalized Sensory Sensitivity Scale (GSS) and/or a body map assessing widespread pain such as the Widespread Pain Index (WPI) [32, 52]. A comprehensive review of the assessment of nociplastic pain and SPACE symptoms, with measure recommendations, was provided by Williams (2018) [53], with additional brief screening measures for affective symptoms detailed by Van den Ende and colleagues (2025) [54]. For trauma-focused assessment, it is important to understand a person’s degree of current stress burden or active distress as well as their overall trauma exposure load [5]. Specific combinations of relevant measures could involve the PCL-PC, CTES/RTES, or ACEs combined with a measure of situational stress [6, 55–57]. If given, providers should be prepared to discuss and refer appropriately as explained below.

A robust review detailing phenotypic-directed approaches to IC/BPS was authored last year in *Current Bladder Dysfunction Reports* [50]. This review emphasized that phenotyping is a work in progress and occurs in “broad brushstrokes,” with many patients having overlapping symptoms [50, 51]. Existing evidence suggests that phenotypic-directed care using multimodal, tailored treatment plans improves outcomes for individuals with complex symptom pictures, which is replicated across cultures [37, 49, 58].

## Management

### Psychological Interventions: CBT, Hypnosis, and Emerging Therapies

Targeted psychological interventions can meaningfully alter the trajectory of LUTS. Cognitive-behavioral therapy (CBT) is feasible in urologic populations. McKernan et al. developed a CBT program specific to IC/BPS, piloted in a RCT of patients with predominantly widespread, refractory pain averaging 14 years in duration [59]. Attendance rates were high, and patients receiving CBT versus an active attention control achieved meaningful improvement at eight weeks that was maintained at six months [59]. The mechanism of improvement was not reduction in anxiety or depression, but rather improved pain coping and self-efficacy, with reduced pain interference [59]. A separate RCT by Yu et al. similarly demonstrated that CBT combined with bladder treatment produced significantly greater perceived improvement in symptoms and anxiety than bladder monotherapy alone (*p* < 0.001 at 12 weeks) [60]. Funada and colleagues (2020) showed that a 4-session CBT is feasible for OAB across a wide range of ages (mean age = 72, range 53–80 years) [61]. Applying CBT to urologic pain and LUTS is expanding in access and awareness, with additional trials underway assessing its potential in reduced-contact, remote, and in-person formats [62, 63].

Clinical hypnosis has also shown promise. In addition to groups demonstrating its feasibility in IC/BPS, McKernan et al. found that individuals with complex pain and LUTS can benefit from hypnosis [61, 64]. After eight hypnosis sessions, participants reported significantly reduced LUTS (*p*=0.006) after accounting for any change in pain, suggesting that hypnosis can improve urinary function independent of pain relief [64]. Recent work suggests hypnosis may improve LUTS via increasing connectivity between brain regions that respond to and inhibit pain [65].

Two emerging psychotherapies warrant attention, though neither has yet been formally tested in urologic populations. Pain Reprocessing Therapy (PRT) targets the threat appraisal system directly, reframing chronic pain as a reversible, brain-generated phenomenon rather than evidence of tissue damage [66]. In a JAMA Psychiatry RCT, 73% of chronic back pain patients were pain-free or nearly pain-free after PRT, with effects lasting at one year [66]. Emotional Awareness and Expression Therapy (EAET) addresses unresolved trauma and stress-related emotions as drivers of chronic pain; in a JAMA Network Open RCT of older veterans, EAET demonstrated superiority over CBT for pain severity with large effect sizes [66]. Both therapies operate on the same threat-sensitization and stress-response pathways described throughout this review, making them logical candidates for future investigation in UCPPS and LUTS populations.

### Multidisciplinary Treatment

While working alongside embedded psychologists is ideal and common in related fields such as gastroenterology, this practice is in its infancy in urology [67]. Multidisciplinary treatment therefore occurs through carefully-timed referrals, offered with appropriate buy-in from patients, given that patients have highly variable adherence to behaviorally-based treatments for LUTS, particularly when self-directed [68]. The American College of Gynecologists (2020) recommends assessment of emotional wellbeing at every visit for CPP [69]. The urology provider has a unique opportunity to provide evidence-based education that enhances a patient’s understanding of their disease mechanisms, connects this to treatment recommendations, and subsequently enhance motivation to engage in the multidisciplinary approaches that would optimally supplement treatment plans.

Phenotype education, particularly in patients with widespread pain present, can help patients understand that their symptoms may be consequences of a sensitized nervous system that is constantly detecting threat, even if there is no ongoing tissue damage. Communicating the veracity of symptoms, the biological basis of symptom contributors like nociplastic pain, and the potential for improvement with treatment can improve buy-in and potential adherence to treatment recommendations at this stage [70, 71].

### Clinical Management and Communication Strategies

Patients living with LUTS, CPP, and UCPPS, often present after years of referral cycles and unsuccessful treatments, with time-to-diagnosis from symptom onset averaging six years [72]. This pattern can leave a wake of frustration, defeat, anger, and mistrust. Adopting a trauma-informed stance and implementing specific language, similar to the kind highlighted in Table 1, can help improve treatment alliance, particularly in the context of prior negative treatment experiences and invalidation commonly experienced in CPP [73]. Maintaining a trauma-informed stance from treatment outset can also enhance perceived control and safety during clinical visits [72].

**Table 1:** Example referral language for providers

Purpose	Example language
State the neurobiologic basis of symptoms	Your pain has a neurobiological basis. Stress and threat sensitivity can amplify pain, inflammation, and bladder symptoms. Treatment may include both medical and behavioral strategies as a part of a multidisciplinary plan to support your care.
Explain stress sensitization	Your system has become sensitized to stress and threat activation, which can also influence inflammation in the body.
Connect treatment to stress regulation	Targeting our stress regulation systems in treatment has shown to help make meaningful change in how we experience pain.
Clarify that symptoms are not “just psychological”	Although anxiety, depression, or other factors may not necessarily be the cause of your pain, they can influence how you experience it.
Explain why referral may help	Addressing anxiety, depression, or stress may ultimately give you additional strategies to manage your pain or urgency, and help you feel more in control of it.
Normalize the interaction between pain and anxiety	Pain can be a little easier to respond to when it is not coupled with anxiety, which can heighten physiological tension. Stress and pain are processed through the same systems in the body. You may have noticed that one can activate the other. Addressing anxiety may indirectly help your pain as well.
Describe why therapy is relevant to symptom flares	Learning how to regulate your nervous system may indirectly help your pain, and reduce your vulnerability to flare episodes.
Position psychotherapy as part of treatment	Therapy is one tool that may help as part of your treatment plan.

### Trauma-Informed Care Strategies

Noting the high proportion of trauma survivors that experience conditions such as pelvic pain, the ACOG (2021) emphasizes adopting universal trauma-informed care (TIC) practices that foster resilience and avoid retraumatization or stigmatization by creating clinical interactions that promote physical and emotional safety [74]. TIC applies directly to the care environment, approach to procedures, and style of communicating with patients.

The American Medical Association (2025) recently created a step-by-step consent guide for potentially painful pelvic procedures, which applies to pelvic exams, diagnostic testing, and in-office procedures in urology [71]. Specific trauma-informed techniques for clinical interactions that may include physical contact (e.g. pelvic exams, urodynamics) have been detailed by McKernan et al. (see Table 5, pp 17) and [5, 75].

Communicating with a TIC approach involves both verbal and non-verbal cues that promote equality and shared decision making [74]. This begins with sitting with a patient on an equal physical level, making eye contact, and discussing the visit plan prior to proceeding with any examination. Providing choice and agency during a visit can enhance perceived safety, with examples including offering a chaperone or support person, reviewing the rationale for an examination, tension points, coping strategies, any adjustments to make together, and making no assumption that a patient will elect to proceed with an exam (particularly in a new consult) [5, 75].

While universal trauma screening is recommended, it should only occur with a plan to respond in kind. Supportive and validating language, coupled with available resources for increased support, help increase provider preparedness for these interactions. Education on the biological basis for symptoms (e.g. the sympathetic nervous system and stress response), threat perceptions, and their connection to urologic health complaints (e.g. pelvic muscle tension) can normalize symptoms and increase motivation for change via non-pharmacological treatment strategies that can calm the nervous system.

### Patient Education

**Fig. 1:**
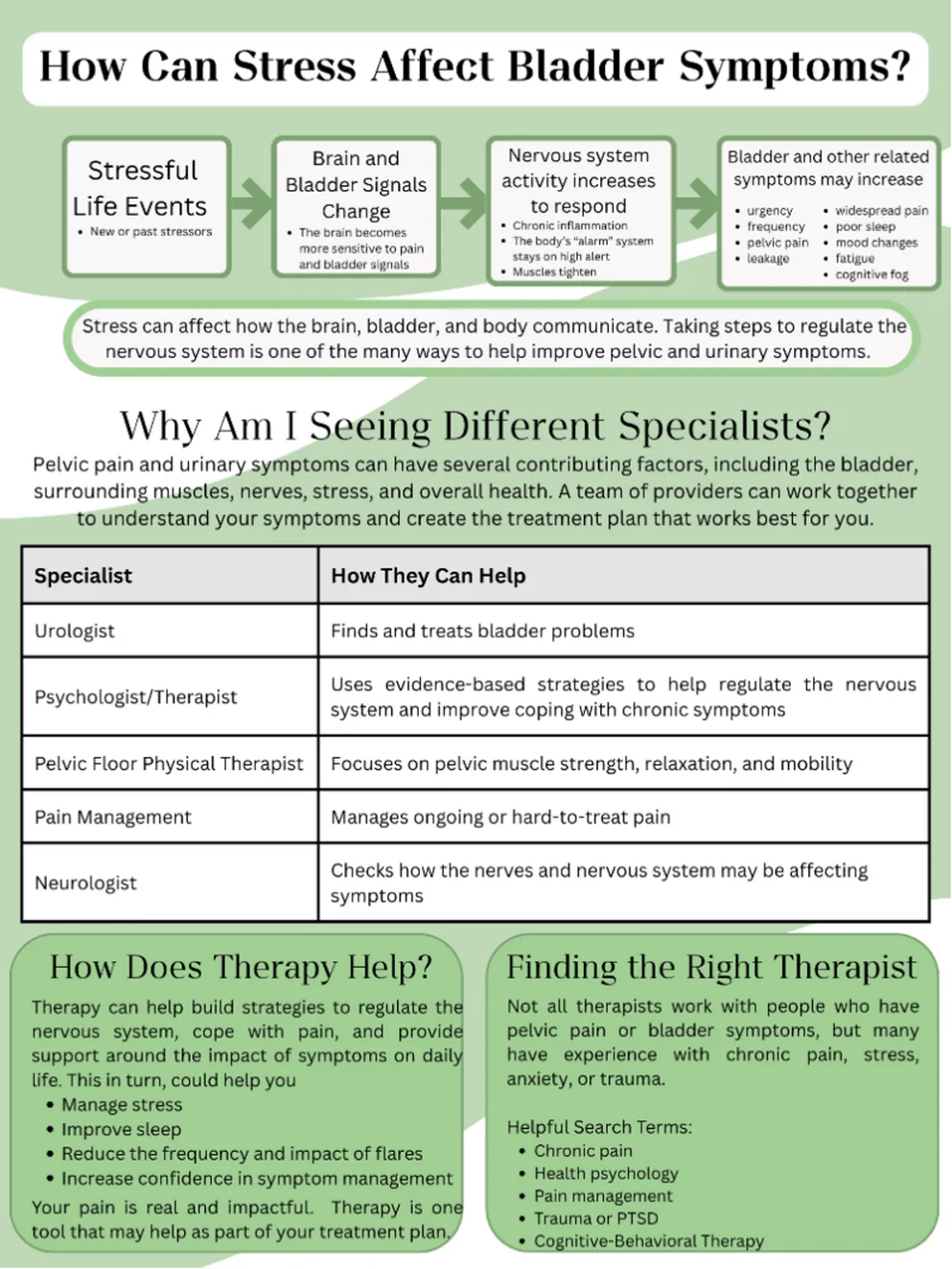
Patient education handout

Finally, it is important that patients leave clinical appointments feeling empowered with reliable and digestible information regarding biopsychosocial concepts and resources available. Qualitative work has shown high demand for such resources characterized by non-stigmatizing explanations, reassurance that treatments are recommended for a specific purpose, and practical guidance for finding the appropriate clinician, whether that be a therapist or specialist, neurologist, pelvic floor physical therapist, or other providers to help further their care [59, 76]. We provide a one-page patient-facing handout describing the neurobiological pathways described here in simple language, explaining the role that psychological and behavioral mechanisms play in addition to appropriate physiological diagnosis and the importance of the multidisciplinary care team (Fig. 1). Pain Neuroscience Education is an additional psychoeducational tool to assist patients in understanding the neurobiology of pain and threat response, which can also be accessed via single session evidence-based treatments for chronic pain such as Empowered Relief [70].

### Future Directions

Several key research priorities emerge for the urologic community. The MAPP Network’s research suggests that simple immune or sensory phenotyping may identify patients unlikely to respond to peripheral therapies. Assessment using both biomarkers and brief clinical measures could enable urologists to triage patients towards tailored interventions earlier in the care pathway. However, no standardized assessment protocol has been validated for urology clinic workflows. Developing and testing a brief screening battery would translate the principles described in this review into routine clinical practice. Moreover, MAPP’s observation that patients with widespread pain respond preferentially to centrally acting therapies, while those with localized pain respond to peripherally directed therapies, needs prospective RCT validation [36, 37]. Another critical gap is the lack of studies evaluating whether integrated biopsychosocial treatment plans combining urologic management, psychological intervention, and pelvic floor PT produce durable improvements in LUTS severity, pain, and quality of life over years rather than weeks to months. Finally, psychotherapies directly targeting threat-appraisal and trauma-processing mechanisms, such as PRT and EAET, have shown promise in other chronic pain populations but have yet to be tested in UCPPS/LUTS.

## Conclusion

This review highlights the growing recognition that LUTS and CPP reflect more than a single anatomical source. Psychosocial factors, including anxiety, depression, OCD, and trauma exposure, are highly prevalent in these populations and are associated with increased symptom severity. Findings from the LURN/MAPP research networks further suggest that many patients exhibit features of nociplastic pain, characterized by widespread pain and sensory sensitivity, which is often accompanied by greater psychosocial complexity. Emerging evidence indicates that simple phenotyping measures can identify clinically meaningful subgroups and help predict treatment response, to inform tailored treatment planning. Accordingly, urologists should adopt a phenotypic, biopsychosocial approach for assessment and management that incorporates psychosocial screening, TIC, multidisciplinary treatment, and patient education.

## Key References

- Schrepf A, Locke K, Moldwin R, Williams DA, Till S, Farrar J, et al. Widespread Pain Moderates the Response to Centrally-Acting Therapies in an Observational Cohort of Patients With Urologic Chronic Pelvic Pain Syndrome: A MAPP Research Network Study. Neurourol Urodyn. 2025;44:1290–5. 10.1002/nau.70,068.
  - ○ This paper provides core evidence supporting phenotype-guided treatments as its a MAPP ATLAS sub-study discussing how patients with widespread pain improved significantly with centrally-directed therapies in comparison to patients with localized pain who responded more favorably to peripherally-directed treatments.
- Farrar JT, Locke KT, Clemens JQ, Griffith JW, Harte SE, Kirkali Z, et al. Widespread pain phenotypes impact treatment efficacy results in randomized clinical trials for interstitial cystitis/bladder pain syndrome: a Multidisciplinary Approach to the Study of Chronic Pelvic Pain network study. Pain. 2025;166:1179–90. 10.1097/j.pain.0000000000003455.
  - ○ This paper highlights the importance of phenotype identification and specific treatment by emphasising that trial outcomes can be masked when researchers fail to take phenotypes into consideration.
- McKernan LC, McGonigle T, Vandekar SN, Crofford LJ, Williams DA, Clauw DJ, et al. A Randomized-Controlled Pilot Trial of Telemedicine-Delivered Cognitive-Behavioral Therapy Tailored for Interstitial Cystitis/Bladder Pain Syndrome. Pain. 2024;165:1748–60. 10.1097/j.pain.0000000000003188.
  - ○ This pilot RCT showed that CBT techniques for IC/BPS could be successfully distributed to patients via telemedicine while maintaining the ability to improve pain coping and self-efficacy. It was one of the first randomized demonstrations that a remote psychological intervention could reach urological pain patients in a meaningful way, further emphasizing our point on the importance of integrating multidisciplinary tools for in urology consultations.

## Data Availability

All data supporting this review can be accessed throughout the paper and through its reference list.

## Declarations

### Use of Artificial Intelligence

All text was independently generated by co-authors and no artificial intelligence was used in generating the content of this article.
